# Efficacy and safety of a novel synergistic drug candidate, CRx-102, in hand osteoarthritis

**DOI:** 10.1136/ard.2007.074401

**Published:** 2007-10-25

**Authors:** T K Kvien, E Fjeld, B Slatkowsky-Christensen, M Nichols, Y Zhang, A Prøven, K Mikkelsen, Ø Palm, A A Borisy, J Lessem

**Affiliations:** 1 Department of Rheumatology, Diakonhjemmet Hospital, Oslo, Norway; 2 Faculty of Medicine, University of Oslo, Norway; 3 Department of Rheumatology, Martina Hansens Hospital, Sandvika, Norway; 4 CombinatoRx Inc., Cambridge, Massachusetts, USA; 5 Department of Rheumatology, Lillehammer Hospital for Rheumatic Diseases, Lillehammer, Norway; 6 Department of Rheumatology, Østfold Hospital, Sarpsborg, Norway

## Abstract

**Objective:**

The novel synergistic drug candidate CRx-102 comprises dipyridamole and low dose prednisolone and is in clinical development for the treatment of immunoinflammatory diseases. The purpose of this clinical study was to examine the efficacy and safety of CRx-102 in patients with hand osteoarthritis (HOA).

**Methods:**

The study was conducted as a blinded, randomised, placebo-controlled trial at four centres in Norway. Eligibility criteria included being of age 30–70 years, at least one swollen and tender joint, a Kellgren–Lawrence (K–L) score of 2 or higher on radiographs, and a score of at least 30 mm pain on the Australian/Canadian Osteoarthritis Hand Index (AUSCAN) visual analogue pain scale (VAS). The primary endpoint was a reduction in pain from baseline to day 42 on the AUSCAN pain subscale. Two-sided p values for the differences in least squares (LS) means adjusted for baseline are presented.

**Results:**

The mean age of the 83 patients with HOA was 60 years and 93% were females. CRx-102 was statistically superior to placebo at 42 days for changes in AUSCAN pain (LS mean −14.2 vs −4.0) and for clinically relevant secondary endpoints (joint pain VAS (−18.6 vs −6.3), patient global VAS (−15.9 vs −4.2)) in the intention to treat population. The most frequently reported adverse event during the study was headache (52% in CRx-102 vs 15% in the placebo group).

**Conclusions:**

The novel synergistic drug candidate CRx-102 demonstrated efficacy by statistically reducing pain compared to placebo in HOA and was generally well tolerated.

Osteoarthritis (OA) is the most common rheumatic joint disease. It is reported to be more prevalent than all other forms of arthritis, and this prevalence seems to be increasing.^1^ The typical clinical manifestations are pain, stiffness and physical disability. Localised loss of hyaline articular cartilage and adjacent bone remodelling are the key structural changes of OA, and local inflammation may also contribute to the pain and joint damage.^2^

Knee OA is the most common form of the disease, followed by hand OA (HOA).^3^ The majority of people aged 55 years and over have radiographic changes of OA in at least one hand joint and approximately one-fifth of this population has symptomatic HOA.^4^ ^5^ The prevalence of HOA increases with age and is higher in females than in males. 4 Recent studies also indicate that the burden of disease for patients with HOA is considerable across a variety of dimensions of health-related quality of life.^6^

Non-steroidal anti-inflammatory drugs (NSAIDs), including selective cyclo-oxygenase-2 (COX-2) inhibitors are important symptom-modifying therapeutic options for patients with OA.^7^^–^10 However, NSAIDs are associated with risk of gastrointestinal adverse events and the selective COX-2 inhibitors have come under special scrutiny because of cardiovascular adverse effects.^11^ ^12^ Similar concerns have recently been raised about the cardiovascular safety of non-naproxen NSAIDs,^13^ and the efficacy of long-term dosing of NSAIDs in knee OA has also been questioned.^14^ Few controlled clinical trials have addressed the efficacy of pharmacological therapies in HOA and, in particular, few controlled studies have included a placebo group.^10^ ^15^ Thus, there is an obvious need to document the efficacy of existing drugs and, in particular, to identify new and effective agents for patients with HOA.^16^

Corticosteroids are a mainstay of effective anti-inflammatory therapy in many clinical settings, but the side effects associated with chronic administration have limited their use in OA to occasional intra-articular administration. It has long been a goal to develop a therapeutic agent with the anti-inflammatory and disease modifying activities of corticosteroids without their associated side effects.^17^ One approach to creating such a therapeutic agent is to develop a drug combination that contains a glucocorticoid and an enhancing agent that pair synergistically to generate a powerful anti-inflammatory effect. Synergistic combinations can have an effect that is greater and more selective than the sum of the activities of the individual components, and thus can provide greater therapeutic benefit with lower toxicity. Synergy is often observed in multi-target therapeutics that modulate the activity of two or more molecular targets to create a novel therapeutic action.^18^ ^19^ In vivo models testing anti-inflammatory combinations containing a low dose steroid have demonstrated a synergistic interaction between the steroid and the enhancing agent that produces an effect equivalent to that of a high dose steroid alone, without indications of high dose steroid side effects.

CRx-102 is one such novel synergistic drug candidate and is in clinical development for the treatment of immunoinflammatory diseases including rheumatoid arthritis (RA) and OA. This drug candidate comprises a combination of a low dose of prednisolone (3 mg) and 200 or 400 mg dipyridamole. According to results from pre-clinical pharmacology experiments, CRx-102 works through a novel mechanism of action by which dipyridamole selectively amplifies prednisolone’s anti-inflammatory and immunomodulatory effects without replicating steroid side effects.^20^^–^22 The objective of the current phase 2 study was to evaluate the efficacy and safety of the novel synergistic drug candidate CRx-102 compared to placebo in patients with HOA over a 6-week dosing period.

## MATERIALS AND METHODS

### Study population

Males and females between the ages of 30 and 70 years with HOA, as defined by the American College of Rheumatology (ACR) criteria,^23^ were enrolled in the study. Additional inclusion criteria included presence of more than one swollen joint and more than one tender joint, a Kellgren–Lawrence (K–L) score of two or more on radiographs and self-reported hand pain that had to be at least 30 mm on the Australian/Canadian Osteoarthritis Hand Index (AUSCAN) visual analogue scales (VAS).^24^ ^25^ All subjects had to sign and date an informed consent. The regional ethics committee evaluated the study, the storage and analyses of data was licensed from the data inspectorate and approval for the collection of biologic material was obtained from the Department of Health.

Subjects who were pregnant, lactating or using hormonal birth control pills as well as subjects with a history of hypersensitivity to corticosteroids and/or dipyridamole, taking bisphosphonates or who had a positive rheumatoid factor test were excluded from the study. Furthermore, subjects who had taken any corticosteroids orally, topically or intra-articularly 3 months prior to enrolment were also excluded. Other exclusion criteria included a history of asthma, HIV infection, hepatitis, currently uncontrolled diabetes, use of statins in a dose that had changed during the prior 3 months, known active infection or a surgical procedure within 30 days of study initiation. Since one of the components of CRx-102 is dipyridamole, patients on warfarin, ticlopidine, clopidogrel or aspirin of more than 81 mg daily were also excluded from entering the study.

### Design and medication

The study was designed as a 6-week randomised, blinded, placebo-controlled four-centre parallel group study. Within 2 weeks of a screening visit, patients who fulfilled the eligibility criteria were randomly assigned to CRx-102 or placebo. Follow-up assessments were performed after 7, 14, 28 and 42 days, with a final safety visit after approximately 56 days.

A total of 83 subjects were randomised 1:1 to a daily dose regimen of either CRx-102 or placebo. CRx-102 for days 1–7 combined 2 mg of prednisolone with 100 mg of dipyridamole at 8am and 1 mg of prednisolone with 100 mg of dipyridamole at 1pm. From days 8–42 CRx-102 combined 2 mg prednisolone with 200 mg of dipyridamole at 8 am and 1 mg of prednisolone with 200 mg dipyridamole at 1pm. Patients in the placebo group received an equal number of tablets, dosed at the same time of day as for the test compound and all containing placebo.

Paracetamol was provided as rescue medication throughout the study at a daily dose of up to 4000 mg, and the usage was recorded. The use of NSAIDs in all subjects was stopped for the duration of the study starting at the initial screening visit.

### Assessments

The AUSCAN^24^ ^25^ was used as the primary assessment tool. Previous studies have demonstrated that this instrument has acceptable reliability, construct validity and responsiveness. The translated Norwegian version has also satisfactory clinimetric properties.^26^ We chose to use the version with responses on VAS to each item. AUSCAN has five items measuring pain, one measuring stiffness and nine measuring physical function. The pain and physical functioning scores were normalised to a 0–100-point scale prior to the analyses.

Additional patient-reported measures included a joint pain VAS (question: how would you describe the intensity of your joint pain during the last 2 days?) and global assessment VAS (question: we ask you to evaluate the activity of your osteoarthritis over the last 2 days. When you take all symptoms into consideration, how will you evaluate your condition?). The patients did not have access to scores from previous visits when they were performing each subsequent assessment.

The patients were clinically examined for vital signs at each visit and each individual finger joint (distal interphalangeal (DIP), proximal interphalangeal (PIP), metacarpophalangeal (MCP) and carpometacarpal (CMC)) on the right and left hand was examined for the presence of joint tenderness, soft tissue swelling, bony enlargement and limited joint motion. A score was calculated using the number of PIP and DIP joints for the presence of each of these four characteristics (ie, score range 0–18 for each).

Sera were frozen and stored and later analysed with a high sensitivity technique to determine levels of C-reactive protein (CRP). Regular blood chemistry, including fasting blood glucose, was recorded. Unsolicited adverse events were also recorded.

### Analyses

AUSCAN pain was the predefined primary endpoint. The sample size was calculated based on an assumed improvement of 20% in AUSCAN pain VAS in the CRx-102 group compared to a 10 % improvement in the placebo-group from baseline to day 42 with an alpha of 0.05%, to achieve 80% power assuming a 15% drop out rate using a one tailed t test for the comparison of the mean changes.

The primary analysis was conducted on the intention to treat (ITT) population that included all patients who took at least one dose of study medication. Secondarily, an analysis was performed in the per-protocol population, which was defined as all subjects who received at least one dose of study medication, had no major protocol violations and had a study drug compliance of at least 75%. The treatment effects were derived from analysis of covariance (ANCOVA) adjusting for the baseline values. These analyses tested the difference (with 95% confidence intervals) between these adjusted mean changes in the active drug compared to the placebo group from baseline to 42 days. The last non-missing post-baseline observation was carried forward (LOCF) to replace subsequent missing values. Two-sided p values for the differences in least square means adjusted for baseline are presented (the study protocol recommended use of one-sided tests, but we found it more appropriate to use two-sided tests, ie, a more conservative approach, in this work). The statistical analyses were performed by the sponsor in collaboration with the principal investigator (TKK).

## RESULTS

**Figure 1:**
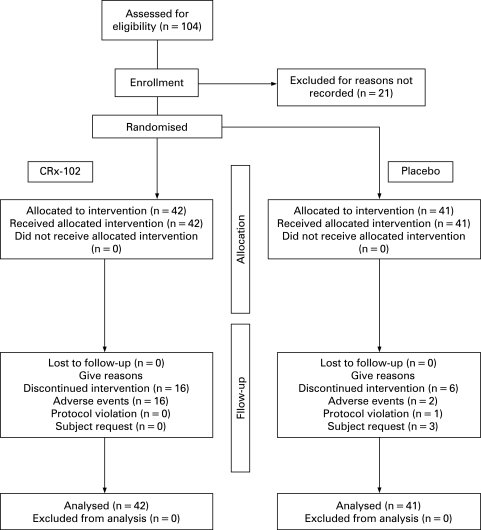
Flow chart of the selection of patients for this study and patient disposition.

A total of 83 patients (77 (93%) females) with a mean (standard deviation (SD)) age of 60.4 (5.2) years were enrolled into the study. A Consolidated Standards of Reporting Trials (CONSORT) flow-chart is shown in fig 1. The study groups were comparable for demographic characteristics (table 1). The patients in the active treatment group had wider OA joint involvement (table 1), but the baseline levels of pain and physical limitations were comparable (table 2).

**Table 1:** Demographic variables and joint involvement (mean (SD)) for continuous variables, percentages for counts)

	CRx-102 (n = 42)	Placebo (n = 41)
Age	61.1 (5.0)	59.6 (5.3)
Female	93	93
Caucasian	100	100
Height, cm	166.0 (6.7)	167.7 (8.2)
Weight, kg	71.1 (12.0)	74.5 (14.6)
Percentage with OA joint involvement:		
Right MTP joint I	36	10
Left MTP joint I	33	10
Lumbar spine	24	17
Cervical spine	19	7
Right hip	17	10
Left hip	10	12
Right knee	19	7
Left knee	17	10
Other joints	17	15
Finger joints: percentage with radiographic grade 2–4 K–L score:		
Minimal (2)	14	12
Moderate (3)	45	32
Severe (4)	40	56

K–L, Kellgren–Lawrence; MTP, metatarsophalangeal; OA, osteoarthritis.

**Table 2:** Baseline mean (SD) values of efficacy variables, adjusted mean changes from baseline to day 42 (least squares mean (standard error of mean)) and treatment effect (mean difference (95% CI) placebo minus CRx-102) in the intention to treat population

	Baseline		Changes		Treatment effect	p Value
	CRx-102 (n = 42)	Placebo (n = 41)	CRx-102	Placebo		
AUSCAN:						
Pain	57.9 (20.2)	60.9 (19.4)	−14.2 (3.0)	−4.0 (3.1)	10.2 (1.6 to 18.7)	0.020
Physical	62.4 (19.5)	67.8 (17.5)	−8.1 (2.7)	−3.6 (2.7)	4.5 (−3.2 to 12.2)	0.246
Stiffness	61.1 (18.0)	64.5 (21.2)	−15.2 (3.2)	−7.7 (3.3)	7.5 (−1.7 to 16.7)	0.108
VAS:						
Joint pain	58.3 (20.1)	62.1 (16.9)	−18.6 (3.3)	−6.3 (3.3)	12.3 (3.0 to 21.5)	0.010
Patient global	58.0 (19.5)	62.3 (17.9)	−15.9 (3.2)	−4.2 (3.3)	11.7 (2.5 to 20.8)	0.013
Lab tests:						
CRP mg/litre	2.5 (2.9)	2.3 (2.2)	−0.2 (0.4)	0.1 (0.4)	0.3 (−0.7 to 1.4)	0.536
Joint counts:						
Tender joints	9.5 (4.7)	9.4 (4.6)	−3.6 (0.7)	−2.4 (0.7)	1.2 (−0.9 to 3.2)	0.258
Soft tissue swelling	5.5 (4.7)	5.0 (4.4)	−2.4 (0.5)	−1.6 (0.5)	0.8 (−0.6 to 2.2)	0.262

AUSCAN, Australian/Canadian Osteoarthritis Hand Index; CRP, C-reactive protein; VAS, visual analogue scale.

**Figure 2:**
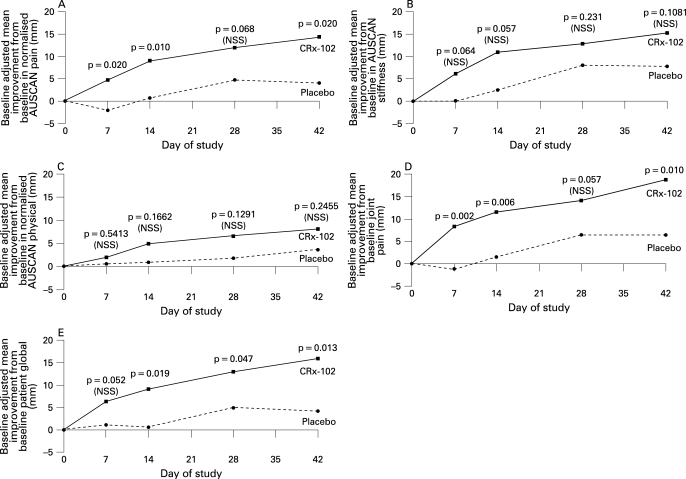
Mean improvements from baseline adjusted for baseline values Australian/Canadian Osteoarthritis Hand Index (AUSCAN) pain (A), AUSCAN stiffness (B), AUSCAN physical (C), pain visual analogue scale (VAS) (D) and global VAS (E) in patients receiving CRx-102 and placebo (intention to treat population) with one-sided p values for the differences of adjusted least square means.

A significant difference (p = 0.020) in the AUSCAN pain score (the primary endpoint) at the end of the study was demonstrated in favour of CRx-102 compared to placebo in the ITT population (table 2). CRx-102 was also statistically superior to placebo at 42 days for joint pain VAS and patient global VAS (table 2). Figure 2 displays how the improvement developed over time and also that the differences between CRx-102 and placebo were discernible after 2 weeks. The comparison between CRx-102 and placebo in the per-protocol population was also consistently in favour of CRx-102 (table 3).

**Table 3:** Baseline mean (SD) values of efficacy variables, adjusted mean changes from baseline to day 42 (least squares mean (SEM)) and treatment effect (mean difference (95% CI) placebo minus CRx-102) in the per-protocol population

	Baseline		Changes		Treatment effect	p Value
	CRx-102 (n = 26)	Placebo (n = 33)	CRx-102	Placebo		
AUSCAN:						
Pain	61.9 (16.6)	63.8 (17.2)	−20.5 (4.1)	−6.2 (3.7)	14.3 (3.2 to 25.5)	0.012
Physical	64.9 (18.9)	70.9 (15.5)	−12.9 (3.7)	−5.9 (3.2)	7.0 (−2.9 to 16.8)	0.061
Stiffness	62.9 (17.4)	67.8 (19.8)	−20.3 (4.4)	−8.3 (3.9)	12.0 (0.2 to 23.9)	0.047
VAS:						
Joint pain	59.8 (19.5)	62.9 (16.7)	−23.5 (4.4)	−6.3 (3.9)	17.2 (5.5 to 28.9)	0.005
Patient global	61.5 (17.5)	62.5 (17.6)	−23.4 (4.0)	−4.6 (3.6)	18.8 (8.1 to 29.5)	0.001
Lab tests:						
CRP mg/litre	2.0 (1.8)	2.3 (2.2)	−0.2 (0.5)	0.4 (0.4)	0.6 (−0.7 to 1.8)	0.364
Joint counts:						
Tender joints	9.6 (4.8)	9.8 (4.7)	−5.0 (1.0)	−2.6 (0.9)	2.4 (−0.3 to 5.0)	0.083
Soft tissue swelling	5.6 (4.6)	4.9 (4.3)	−3.1 (0.6)	−1.9 (0.5)	1.3 (−0.3 to 2.8)	0.116

AUSCAN, Australian/Canadian Osteoarthritis Hand Index; CRP, C-reactive protein; VAS, visual analogue scale.

The tender and swollen joint counts of the 18 PIP and DIP joints were numerically improved in the CRx-102 group compared to placebo (table 2) and the group differences approach statistical significance in the per-protocol population (table 3). The counts of joints with limited motion and bony swelling did not change during the study (data not shown).

**Figure 3:**
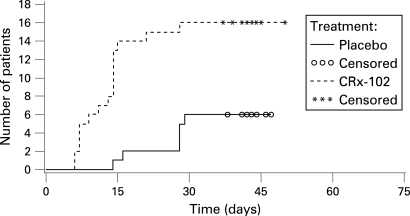
Time from dose 1 to withdrawal from the treatment (intention to treat (ITT) population, Kaplan–Meier plot).

The proportions of patients reporting at least one adverse event in the CRx-102 and placebo groups were 64% and 32%, respectively. The most common adverse event in both groups was headache, which was more frequently reported in the CRx-102 group (52%) than in the placebo group (15%). A total of 21% of the patients in the CRx-102 group also reported nausea, versus none in the placebo group (table 4). No serious adverse events were reported in the CRx-102 group. Discontinuation occurred more often in the CRx-102 (n = 16, 38%) than in placebo group (n = 6, 15%) (fig 1) and was mostly due to headache. Most of the discontinuations occurred early in the study (fig 3).

**Table 4:** Most commonly reported adverse events (AE) (⩾5% of total subjects) in the intention to treat (ITT) population (no. of patients (%))

	CRx-102 (n = 42)	Placebo (n = 41)	Total (n = 83)
Subjects with at least one AE	27 (64)	13 (32)	40 (48)
Headache	22 (52)	6 (15)	28 (34)
Nausea	9 (21)	0	9 (11)

## DISCUSSION

HOA is a frequent disease in people more than 60 years of age and imparts a considerable disease burden, on the individual^6^ and in society.^2^ However, few studies have formally addressed the efficacy of symptom-modifying drugs in HOA.^10^ ^15^ This phase 2 study demonstrated that CRx-102, a combination of a low dose prednisolone and a titrated dose of dipyridamole, was superior to placebo across a variety of patient-reported measures.

In HOA the DIP and PIP joints as well as the first carpometacarpal (CMC) joint are particularly affected,^27^ but patients with HOA tend to have involvement of multiple joints, a condition that is often referred to as generalised OA.^28^ The joints most frequently involved in this study population were the first metatarsophalangeal (MTP) joint, the knee and additionally the lumbar and cervical spine (table 1). We do not know how CRx-102 influenced these other joint areas, since we were not measuring low back pain, pain in the big toe or were not using the Western Ontario and McMaster Universities (WOMAC) index,^29^ a specific measure of knee and hip OA. However, the question on joint pain did not specifically address pain in the finger joints and the clear differentiation between CRx-102 and placebo for this measure may indirectly support an efficacy that goes beyond the finger joints.

Inflammation has been recognised as a feature of the disease process in OA.^30^^–^33 A synergistic drug candidate such as CRx-102 comprises two components that are designed to act synergistically through multiple pathways, providing a novel therapeutic effect that neither component can achieve on their own. CRx-102 has been shown to have strong anti-inflammatory effects in preclinical assays with a greater average percentage inhibition of tumour necrosis factor alpha (TNFα) and interleukin 1 (IL1) release from human blood buffy coat cells than the inhibition seen with each of the single agents alone.^20^ Similarly, in the rat model of lipopolysaccharide (LPS)-induced TNFα release, the inhibition of release was greater with CRx-102 than the individual components.^21^ This finding was replicated in the rat models of adjuvant and collagen-induced arthritis.^21^

The preclinical studies on the mechanism of action of CRx-102 indicate that CRx-102 acts through multiple molecular pathways to create a synergistic immunomodulatory effect without amplifying traditional glucocorticoid-associated side effects. Additionally, the components of CRx-102 affect the activity of key transcription factors, but not their nuclear localisation nor does it increase glucocorticoid receptor translocation or transcription from positive glucocorticoid response element promoters relative to low dose prednisolone on its own.^22^ Dipyridamole may contribute to the action of CRx-102 by increasing cAMP in part though inhibition of phosphodiesterases relevant to inflammation, as well as through modulating adenosine transport resulting in increased extra-cellular endogenous adenosine.^34^ Adenosine can suppress the release of TNFα from activated monocytes and macrophages, but a small placebo-controlled study was unable to demonstrate clinical improvement following treatment with dipyridamole.^35^

This study used the pain dimension in AUSCAN as the primary endpoint, but also included an assessment of two other core measures, function and patient global assessment.^36^ ^37^ AUSCAN has been developed as a disease-specific patient-reported measure in hand OA. Development of the AUSCAN HOA index was based on the questions and experience from the WOMAC^38^ as well as information from patient interviews. 24 AUSCAN was later validated in separate studies^25^ and has also been validated in the Norwegian language.^26^ Consistent with the present findings, Allen *et al*^39^ showed that the AUSCAN index can reliably measure changes in pain, stiffness and function, thereby providing a meaningful endpoint for clinical trials in HOA. For feasibility reasons we chose not to include other hand indexes that are alternatives to AUSCAN in the assessment of HOA.^40^ ^41^

The patients had to stop treatment with NSAIDs and paracetamol at the screening visit, but a disease flare was not required for inclusion in the study. We unfortunately did not capture detailed information about ongoing medication at the screening visit, and we do not have detailed data on the changes in efficacy endpoints from screening to randomisation.

In rheumatoid arthritis, several composite disease measures have been developed over the past years, and these are most often based on joint counts. Similar composite disease specific measures do not exist in HOA. We examined the number of finger joints with tenderness, soft tissue swelling, limited motion and bony swelling at baseline and during follow-up. It is reasonable to assume that tenderness and soft tissue swelling at least in part reflect the inflammatory component of the disease. Joint counts were numerically improved in the CRx-102 treatment group compared to the placebo group (tables 3 and 4). The number of joints with bony swelling did not change during the study; this endpoint was not expected to be influenced by anti-inflammatory therapy. More research is needed to address the development of joint count-indices for HOA that are valid, reliable and responsive.

Headache was the most commonly reported adverse event in this trial and was most frequently observed during the first days of treatment (table 4 and fig 3). This type of headache has previously been associated with dipyridamole administration^42^ and also with other cardiovascular pharmaceutical products with vasodilatating properties. As in the current study, these headaches typically occur during the first days of treatment.^42^ This early and high withdrawal rate is a potential limitation of CRx-102. Thus, formulation development is necessary to optimise the synergistic benefits of CRx-102 demonstrated in this and other phase 2 studies and to minimise the observed incidence of headache. The objective of the formulation should be to deliver pulsed-doses of prednisolone with concurrently releasing dipyridamole at a rate that maintains the anti-inflammatory synergy and minimises the vasodilator effects that are known to cause headaches.

In summary, preclinical studies have supported that the synergistic drug candidate CRx-102, comprised of dipyramidole and low dose prednisolone, has biological effects that exceed the effects of each individual component. This placebo-controlled phase 2 study suggests that the combination is effective in patients with HOA. Follow-up studies should be initiated to compare the clinical effects of CRx-102 versus the individual components in HOA and other rheumatic joint diseases.
